# Supplementary material for: The phage shock protein (PSP) envelope stress response: discovery of novel partners and evolutionary history
Source: mSystems. 2024 May 29;9(6):e00847-23. doi: 10.1128/msystems.00847-23 (PMC11237479; doi:10.1128/msystems.00847-23)
Supplement: Supplemental Material — Fig. S1 and S2; Tables S1-S4; supplemental text. [file msystems.00847-23-s0001.pdf]

The Phage-shock-protein (PSP) Envelope Stress Response: Discovery of Novel Partners and Evolutionary History

Janani Ravi[1,2]*, Vivek Anantharaman[3], Samuel Zorn Chen[4], Evan Pierce Brenner[1], Pratik Datta[2], L Aravind[3*], Maria Laura Gennaro[2]*.
[1]Department of Biomedical Informatics, University of Colorado Anschutz Medical Campus, Aurora, CO, USA; [2]Public Health Research Institute, Rutgers University, Newark, NJ, USA; [3]National Center for Biotechnology Information, National Institutes of Health, Bethesda, MD, USA; [4]Computer Science Engineering Program, Michigan State University, East Lansing, MI, USA. *Corresponding authors: janani.ravi@cuanschutz.edu; aravind@nih.gov; marila.gennaro@rutgers.edu

Table of Contents

Supplementary Figures and Tables

Supplementary Figures

Figure S1. Mutual exclusion phyletic pattern of PspM, Thioredoxin

Figure S2. PspA maximum likelihood gene tree with bootstrap values by RAxML-NG.

Supplementary Tables

Table S1. Summary of PSP partner domains (query proteins)

Table S2. Summary of novel PSP partner domains

Table S3. Domain architectures, genomic contexts, and lineages of representative PspA/Snf7 homologs. (next pages)

Table S4. Domain architectures, genomic contexts, and lineages of representative homologs of PSP cognate partner domains. (next pages)

Supplementary Text

1. Domain definitions

1.1. LiaI-LiaF-TM and Toastrack domains

1.2 PspM and PspN domains

1.3 PspAA and PspAB domains

2. Novel PSP associations

2.1 PspA/Snf7 domain architectures

2.2 Vps4 and AAA$^+$-ATPases

2.3 PspA with PspM or Thioredoxin

2.4 Novel contexts containing Toastrack

Toastrack and TM domains

Toastrack and transcription factors

References

Supplementary Figures and Tables

All PSP results (data summarizations and visualizations) can be accessed via our easy-to-use interactive web app: https://jravilab.org/psp.

Supplementary Figures

Figure S1. Mutual exclusion phyletic pattern of PspM, Thioredoxin

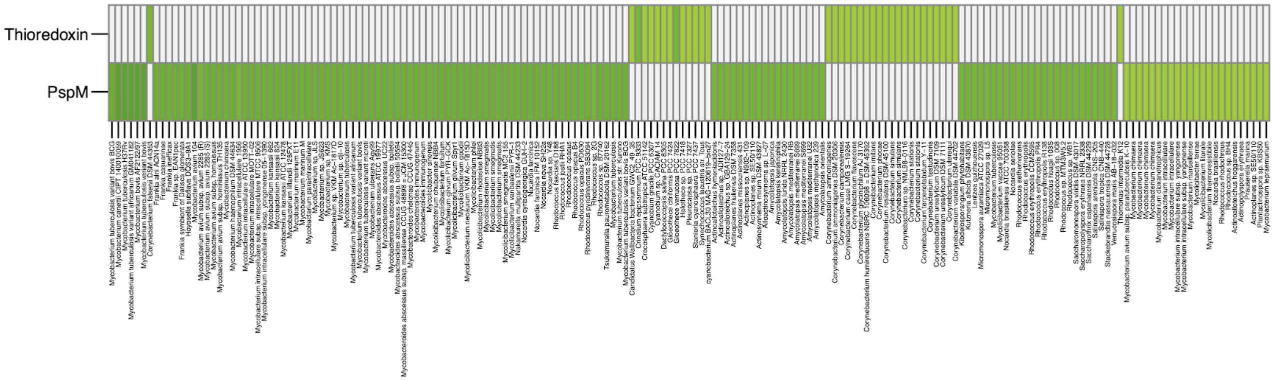

Figure S2. PspA maximum likelihood gene tree with bootstrap values by RAxML-NG.

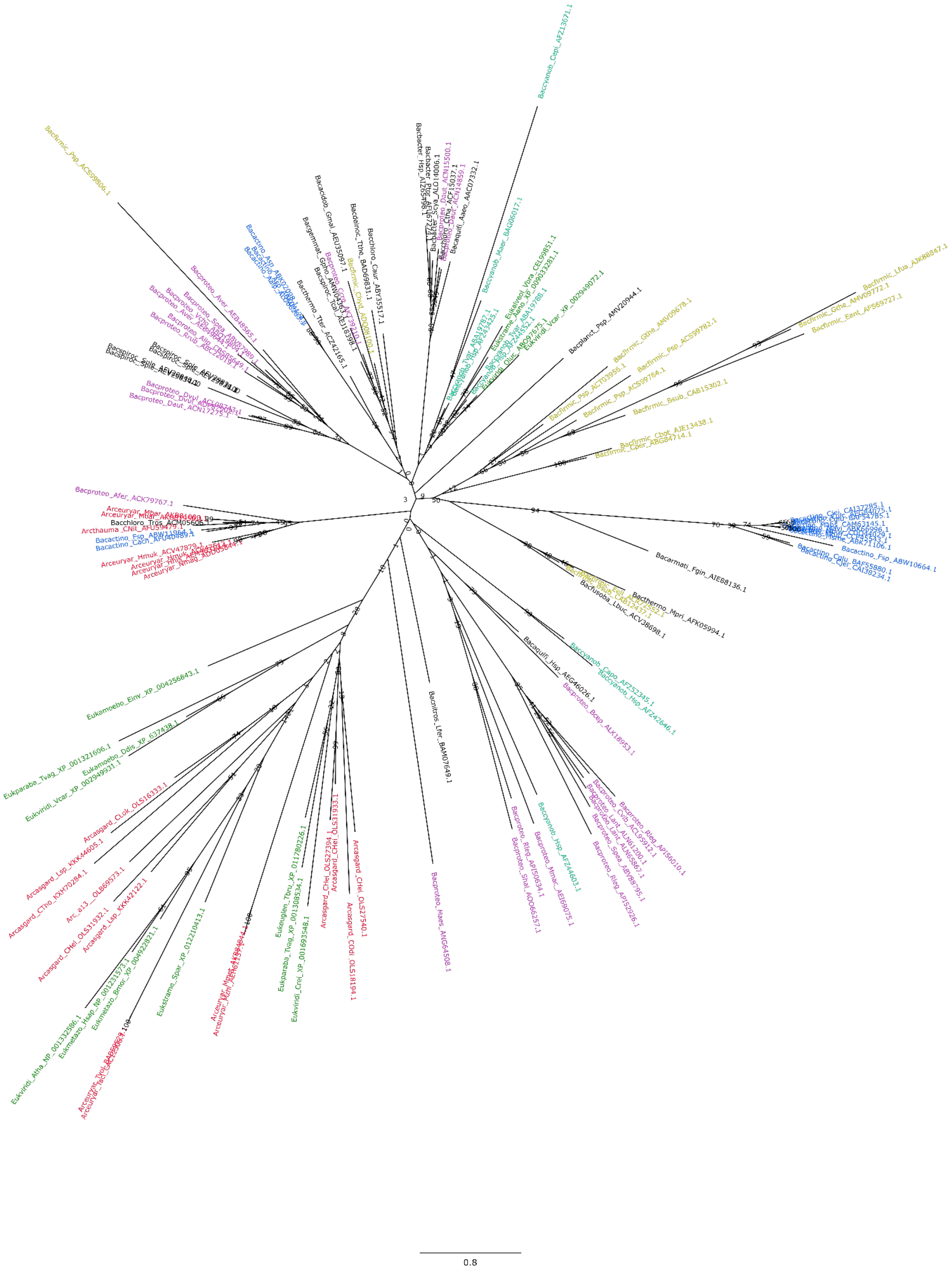

Supplementary Tables

Table S1. Summary of PSP partner domains (query proteins)

Domain Name	Old Name/Aliases	Pfam	PDB
PspA	PspA_IM30<br>LiaH, Vipp1, YjfJ, YdjF	PF04012	4WHE
Snf7		PF003357	5FD7
PspA/Snf7	PspA/ESCRT-III	CL0235	3FRV
Toastrack	DUF4097/DUF2154/DUF2807<br>LiaF/LiaG, YvlB, YthC, Toastrack_N (N-terminal region of Toastrack; PF17115)	PF13349/<br>PF09922/<br>PF10988/PF17115	4QRK/<br>4OPW
LiaI-LiaF-TM	DUF2157<br>LiaI/LiaF	PF09925	
PspM	Rv2743c		
PspN	Rv2742c		
PspN_N	N-terminal region of PspN		
DUF3046	C-terminal region of PspN	PF11248	
PspB		PF06667	
PspC	YthA/YthB, YvlC	PF04024	

Table S2. Summary of novel PSP partner domains

Domain Name	Old Name/Aliases	Pfam	PDB
HAAS	DUF1700 alpha-helical/<br>DUF1129/Yip1/<br>DUF1048	PF08006/PF06570/<br>PF04893/PF06304	2O3L
SHOCT-bihelical	DUF1707, SHOCT	PF08044/PF09851	
PspAA	PspA-associated domain A		
PspAB	PspA-associated domain B		
Vps4-AAA-ATPase/<br>Classical-AAA-ATPase		PF08432/<br>PF00004	5FVK/<br>3U5Z
MIT		PF04212	5FVK

Thioredoxin		PF00085	2OE3
ClgR-HTH	XRE-HTH/HTH_3, ClgR, cHTH	PF01381	6IRP
TM-Flotillin dyad	Flot	PF15975	
Band-7	Band_7	PF01145	3BK6
Spermine synthase	Spermine_synth	PF01564	6O63
YjfL-TM(s)	DUF350	PF03994	
CesT_Tir	CesT	PF05932	1TTW
CesT_Tir-DUF2170	DUF2170	PF09938	
TPM_phosphatase		PF04536	4OA3
SHOCT-like	DUF1707	PF08044	
Caspase	Peptidase_C14	PF00656	3UO8
PadR-like_wHTH PadR-wHTH	PadR-HTH/PadR	PF03551	1XMA
RHH	TraJ-RHH/RHH_1	PF01402	3OD2
SIGMA-HTH			
GerE-HTH/ DUF2089-HTH	GerE/ DUF2089	PF00196/PF09862	2JPC
GNTR-HTH	GntR	PF00392	4R1H

Table S3. Domain architectures, genomic contexts, and lineages of representative PspA/Snf7 homologs. (next pages)

Table S4. Domain architectures, genomic contexts, and lineages of representative homologs of PSP cognate partner domains. (next pages)

Table S3: Representative PspA/Snf7 homologs

Gene, Lineage information and Genomic Contexts grouped by Domain Architectures

<table>
<tr><th colspan="2">Gene Info</th><th colspan="2">Lineage Info</th><th></th></tr>
<tr><th>AccNum</th><th>GeneName</th><th>Species</th><th>Lineage</th><th>GenContext</th></tr>
<tr><td colspan="5">MIT+Vps4-AAA-ATPase</td></tr>
<tr><td>CKH37208.1</td><td>ftsH_1</td><td>Mycolicibacterium smegmatis</td><td>bacteria>actinobacteria</td><td>MIT+Vps4-AAA-ATPase-></td></tr>
<tr><td>ACB74714.1</td><td>Oter_1429</td><td>Opitutus terrae PB901</td><td>bacteria>PVC_group>verrucomicrobia</td><td>MIT+Vps4-AAA-ATPase->TPR+TM(s)-></td></tr>
<tr><td colspan="5">NlpC+PspA</td></tr>
<tr><td>AFZ52345.1</td><td>Cyan10605_0189</td><td>Cyanobacterium aponinum PCC 10605</td><td>bacteria>cyanobacteria</td><td>NlpC+PspA-></td></tr>
<tr><td colspan="5">PspA</td></tr>
<tr><td>AKJ06548.1</td><td>AA314_08174</td><td>Archangium gephyra</td><td>bacteria>proteobacteria>deltaproteobacteria</td><td><-ABC-ATPase||Glycos_trans_3N+Glycos_transf_3+PYNP_C->X(s)->PspA->SIG+PBPB->SIG+TM+Snf7->||<-X||SIG+DUF3352->ABC-ATPase->SIG+TM(s)->SIG+TM(s)-></td></tr>
<tr><td>AEY64321.1</td><td>Clo1100_0028</td><td>Clostridium sp BNL1100</td><td>bacteria>firmicutes</td><td><-ParA-Soj-PloopNTPase||X(s)->GNTR-HTH->inactive-Classical-AAA+Classical-AAA->PspA->ACET-></td></tr>
<tr><td>ANQ40502.1</td><td>BAR24_02900</td><td>Gluconobacter oxydans</td><td>bacteria>proteobacteria>alphaproteobacteria</td><td><-PspF-NtrC-AAA+FIS-HTH||PspA->PspB->TM+Toastrack->TM+Toastrack->SIG+TM(s)->ABC-ATPase-></td></tr>
<tr><td>AOL22920.1</td><td>Ga0102493_111899</td><td>Erythrobacter litoralis</td><td>bacteria>proteobacteria>alphaproteobacteria</td><td><-PspF-NtrC-AAA+FIS-HTH||X->PspA->PspB->PspC+PspB->SIG+PspB->SIG+PspB->SIG+PspB->SIG+PspB->PspB-></td></tr>
<tr><td>BAB38581.1</td><td>yjfJ</td><td>Escherichia coli O157:H7 str Sakai</td><td>bacteria>proteobacteria>gammaproteobacteria</td><td>CesT_Tir-DUF2170->PspA->DUF2491->YjfL-TM+TM(s)->LipoSIG+DUF1190->SpermGS-ATPgrasp-></td></tr>
<tr><td>AMJ95269.1</td><td>AVL56_13815</td><td>Alteromonas addita</td><td>bacteria>proteobacteria>gammaproteobacteria</td><td>CesT_Tir-DUF2170->PspA->Ion_trans_2+TrkA_N+TrkA_C->DUF2491->YjfL-TM+TM(s)->LipoSIG+DUF1190->SpermGS-ATPgrasp-></td></tr>
<tr><td>ABK71106.1</td><td>MSMEG_2695</td><td>Mycolicibacterium smegmatis MC2 155</td><td>bacteria>actinobacteria</td><td>ClgR-HTH->PspA->PspM->||<-X(s)->DUF3046-></td></tr>
<tr><td>CCP45543.1</td><td>35kd_ag</td><td>Mycobacterium tuberculosis H37Rv</td><td>bacteria>actinobacteria</td><td>ClgR-HTH->PspA->PspM->PspN_N+DUF3046->||<-X(s)->DUF3046-></td></tr>
<tr><td>AOS62694.1</td><td>TL08_09395</td><td>Actinoalloteichus hymeniacidonis</td><td>bacteria>actinobacteria</td><td>ClgR-HTH->UA74_09550-lowcomplexity->PspA->PspM-></td></tr>
<tr><td>ANX06812.1</td><td>AS891_06225</td><td>Bacillus subtilis subsp subtilis</td><td>bacteria>firmicutes</td><td>LiaI-LiaF-TM->PspA->TM+Toastrack->LiaI-LiaF-TM+Toastrack->SIG+TM+HAMP+HISKIN->REC+wHTH-></td></tr>
<tr><td>AAN56746.1</td><td>SO_3765</td><td>Shewanella oneidensis MR1</td><td>bacteria>proteobacteria>gammaproteobacteria</td><td>LipoSIG+Ctha_1186+Low-comp->YjfL-TM+TM(s)->SIG+TM(s)+Spermine_synth->CesT_Tir-DUF2170->PspA->DUF4178->RHH-></td></tr>
<tr><td>AFZ14666.1</td><td>Cri9333_3857</td><td>Crinalium epipsammum PCC 9333</td><td>bacteria>cyanobacteria</td><td>PspA->PspA->Thioredoxin-></td></tr>
<tr><td>AAM04874.1</td><td>MA_1460</td><td>Methanosarcina acetivorans C2A</td><td>archaea>euryarchaeota</td><td>PspA->PspAA-></td></tr>
<tr><td>CAB51252.1</td><td>SCO2168</td><td>Streptomyces coelicolor A32</td><td>bacteria>actinobacteria</td><td>PspA->PspAA->SIG+TM(s)+HISKIN->REC+wHTH-></td></tr>
<tr><td>ABW11964.1</td><td>Franean1_2534</td><td>Frankia sp EAN1pec</td><td>bacteria>actinobacteria</td><td>PspA->PspAA->TM(s)+Metallopeptidase+TM(s)->PspAB-></td></tr>
<tr><td>AEN65073.1</td><td>Entas_2342</td><td>Enterobacter soli</td><td>bacteria>proteobacteria>gammaproteobacteria</td><td>PspA->PspB->PspC+PspB->PspD->DO-GTPase2->TM(s)+IIGP1->PspF-NtrC-AAA+FIS-HTH-></td></tr>
<tr><td>ANW39986.1</td><td>A9L45_10880</td><td>Escherichia coli O157:H7</td><td>bacteria>proteobacteria>gammaproteobacteria</td><td>PspA->PspB->PspC+PspB->PspD->PspE-SIG+RHOD-CDC25-></td></tr>
<tr><td>ANX09535.1</td><td>AS891_20665</td><td>Bacillus subtilis subsp subtilis</td><td>bacteria>firmicutes</td><td>PspA->ZnR(s)+TM->SIG+TPM_phosphatase+TM->Band-7+ZnR-></td></tr>
<tr><td>CBH24266.1</td><td>SRM_01345</td><td>Salinibacter ruber M8</td><td>bacteria>FCB_group>bacteroidetes</td><td>Ribosomal_L31->Glycos_trans_3N+Glycos_transf_3+PYNP_C->PspA->Rmar_0091-Coiled-coil->SIG+TM+Snf7->||<-X<-MoaC</td></tr>
<tr><td>AKX93460.1</td><td>MOTHE_c06560</td><td>Moorella thermoacetica</td><td>bacteria>firmicutes</td><td>SIG+SHOCT-bihelical->TM(s)+Metallopeptidase+TM(s)->PspAB->PspA->PspAA-></td></tr>
<tr><td>APB74393.1</td><td>PPYC2_05025</td><td>Paenibacillus polymyxa</td><td>bacteria>firmicutes</td><td>SIG+TM(s)->LiaI-LiaF-TM->PspA->PspC+Coiled-coil->PspA->SIG+TM+Toastrack->SIG+TM+HISKIN->REC+wHTH-></td></tr>
<tr><td>AKK09942.1</td><td>CTEST_12695</td><td>Corynebacterium testudinoris</td><td>bacteria>actinobacteria</td><td>Thioredoxin->PspA-></td></tr>
<tr><td>ANH61663.1</td><td>I597_2772</td><td>Dokdonia donghaensis DSW1</td><td>bacteria>FCB_group>bacteroidetes</td><td>TM(s)->PadR-like-wHTH->HAAS+PspC+LiaI-LiaF-TM+Toastrack->SIG+NTF2->TM+Toastrack->||<-CHTH+Protease||CesT_Tir->PspA->YqiJ-YuaF-SIG+TM(s)->SIG+Band-7+Coiled-coil+TM-Flotillin->Betapropeller+Coiled-coil+AAA-ATPase-></td></tr>
<tr><td>AKB54760.1</td><td>MSBRM_1762</td><td>Methanosarcina barkeri MS</td><td>archaea>euryarchaeota</td><td>TM(s)+Metallopeptidase+TM(s)->PspAB->||<-X(s)->PspA->PspAA-></td></tr>
<tr><td colspan="5">PspA(s)</td></tr>
<tr><td>BAG06017.1</td><td>MAE_61950</td><td>Microcystis aeruginosa NIES843</td><td>bacteria>cyanobacteria</td><td>PspA->PspA->PspA(s)-></td></tr>
<tr><td colspan="5">PspA+PspAA</td></tr>
<tr><td>ACU53894.1</td><td>Afer_0955</td><td>Acidimicrobium ferrooxidans DSM 10331</td><td>bacteria>actinobacteria</td><td>PspA+PspAA-></td></tr>
<tr><td colspan="5">PspAB</td></tr>
<tr><td>AAZ55047.1</td><td>Tfu_1009</td><td>Thermobifida fusca YX</td><td>bacteria>actinobacteria</td><td><-TIMbarrel<-X||PspA->PspAA->TM(s)+Metallopeptidase+TM(s)->PspAB-></td></tr>
<tr><td colspan="5">SIG+MMPL+Snf7+MMPL</td></tr>
<tr><td>CAM62382.1</td><td>MAB_2301</td><td>Mycobacteroides abscessus ATCC 19977</td><td>bacteria>actinobacteria>actinobacteria</td><td>Mycobact_memb->SIG+MMPL+Snf7+MMPL-></td></tr>
<tr><td colspan="5">SIG+TM+Snf7</td></tr>
<tr><td>OGG56892.1</td><td>A3F84_10925</td><td>Candidatus Handelsmanbacteria bacterium RIFCSPLOWO2_12_FULL_64_10</td><td>bacteria</td><td>FAD_binding_5+CO_deh_flav_C->X(s)->PspA->SIG+PBPB+OmpA->SIG+TM(s)->ABC-ATPase->SIG+TM+Snf7->inactive-Classical-AAA+Classical-AAA-></td></tr>
<tr><td colspan="5">Snf7</td></tr>
<tr><td>OLS27540.1</td><td>HeimC3_03190</td><td>Candidatus Heimdallarchaeota archaeon LC_3</td><td>archaea>asgard_group</td><td>MIT+Vps4-AAA-ATPase->||<-X(s)->Snf7->Snf7->MIT+Vps4-AAA-ATPase->ESCRT-II-></td></tr>
<tr><td>CBY21170.1</td><td>GSOID_T00008924001</td><td>Oikopleura dioica</td><td>eukaryota>metazoa>chordata</td><td>Snf7->Snf7->Snf7-></td></tr>
</table>

More information available on our webapp.

Table S4: Representative homologs of Psp cognate partner domains

Gene, Lineage information and Genomic Contexts grouped by Domain Architectures

Gene Info		Lineage Info		
AccNum	GeneName	Species	Lineage	GenContext
DUF4178				
AAN56747.1	SO_3766	Shewanella oneidensis MR1	bacteria>proteobacteria>gammaproteobacteria	LipoSIG+Ctha_1186+Low-comp->YjfL-TM+TM(s)->SIG+TM(s)+Spermine_synth->CesT_Tir-DUF2170->PspA->DUF4178->RHH->
HAAS+FTSW_RODA_SPOVE				
CAC98500.1	lmo0421	Listeria monocytogenes EGDe	bacteria>firmicutes	SIGMA-Factor->PadR-like-wHTH->HAAS+FTSW_RODA_SPOVE->
HAAS+MacB_PCD+FtsX+MacB_PCD+FtsX				
ACO32024.1	ACP_2125	Acidobacterium capsulatum ATCC 51196	bacteria>acidobacteria	PadR-like-wHTH->HAAS+MacB_PCD+FtsX+MacB_PCD+FtsX->
HAAS+Pentapeptide				
AOH56696.1	ABE28_020195	Bacillus muralis	bacteria>firmicutes	PadR-like-wHTH->HAAS+Pentapeptide->
LiaI-LiaF-TM				
APB74392.1	PPYC2_05020	Paenibacillus polymyxa	bacteria>firmicutes	SIG+TM(s)->LiaI-LiaF-TM->PspA->PspC+Coiled-coil->PspA->SIG+TM+Toastrack->SIG+TM+HISKIN->REC+wHTH->
LiaI-LiaF-TM+Toastrack				
ABD83157.1	Sde_3902	Saccharophagus degradans 240	bacteria>proteobacteria>gammaproteobacteria	LiaI-LiaF-TM+Toastrack->SIG+TM(s)+HISKIN->REC+wHTH->
AFH48155.1	IALB_0443	Ignavibacterium album JCM 16511	bacteria>ignavibacteriae	LiaI-LiaF-TM+Toastrack->SIG+TM(s)+HISKIN->REC+wHTH->X(s)->TM->
AGK93623.1	LA14_0400	Lactobacillus acidophilus La14	bacteria>firmicutes	LytTR->LiaI-LiaF-TM+Toastrack->
CAL82154.1	CBO0601	Clostridium botulinum A str ATCC 3502	bacteria>firmicutes	LytTR->LiaI-LiaF-TM+Toastrack->
PspC				
CAB15516.1	yvlC	Bacillus subtilis subsp subtilis str 168	bacteria>firmicutes	yvlA-SIG+TM(s)->SHOCT-like+Toastrack->PspC->SIG+TM(s)->||<-SIG+TM(s)
PspC+LiaI-LiaF-TM				
ABY34522.1	Caur_1294	Chloroflexus aurantiacus J10fl	bacteria>chloroflexi	PspC+LiaI-LiaF-TM->
PspC+LiaI-LiaF-TM+HISKIN				
AIJ13865.1	SLIV_14405	Streptomyces lividans TK24	bacteria>actinobacteria	<-PspC+TM(s)+Toastrack||PspC+LiaI-LiaF-TM+HISKIN->REC+wHTH->
PspC+LiaI-LiaF-TM+TM(s)+Toastrack				
ACV77657.1	Namu_1253	Nakamurella multipartita DSM 44233	bacteria>actinobacteria	<-REC+wHTH<-PspC+LiaI-LiaF-TM+HISKIN||PspC+LiaI-LiaF-TM+TM(s)+Toastrack->X->||<-SIG+TM(s)
PspC+TM(s)+Toastrack				
AIJ13866.1	SLIV_14410	Streptomyces lividans TK24	bacteria>actinobacteria	<-REC+wHTH<-PspC+LiaI-LiaF-TM+HISKIN||PspC+TM(s)+Toastrack->
SHOCT-bihelical+Toastrack				
CCP43715.1	Rv0966c	Mycobacterium tuberculosis H37Rv	bacteria>actinobacteria	SHOCT-bihelical+Toastrack->
CAB88834.1	SCO2893	Streptomyces coelicolor A32	bacteria>actinobacteria	TM(s)->TM(s)->ABC-ATPase->SHOCT-bihelical+Toastrack->
SHOCT-like+Toastrack				
CAB15517.1	yvlB	Bacillus subtilis subsp subtilis str 168	bacteria>firmicutes	yvlA-SIG+TM(s)->SHOCT-like+Toastrack->PspC->SIG+TM(s)->||<-SIG+TM(s)
SIG+LiaI-LiaF-TM+TM+Toastrack				
AEU34960.1	AciX8_0610	Granulicella mallensis MP5ACTX8	bacteria>acidobacteria	SIGMA-Factor->anti-sigma-ZF+TM->TM+LiaI-LiaF-TM->SIG+LiaI-LiaF-TM+TM+Toastrack->
SIG+LiaI-LiaF-TM+Toastrack				
CAB15300.1	liaF	Bacillus subtilis subsp subtilis str 168	bacteria>firmicutes	LiaI-LiaF-TM->PspA->TM+Toastrack->LiaI-LiaF-TM+Toastrack->SIG+TM+HAMP+HISKIN->REC+wHTH->
SIG+TM(s)+Toastrack				
ABD31150.1	SAOUHSC_02100	Staphylococcus aureus subsp aureus NCTC 8325	bacteria>firmicutes	SIG+TM(s)+Toastrack->SIG+TM(s)+HISKIN->REC+wHTH->||<-SIG+TM(s)
TM+DUF4178				
CCP45393.1	Rv2597	Mycobacterium tuberculosis H37Rv	bacteria>actinobacteria	TM+DUF4178->DUF2617->SIG+DUF4247->YjfL-TM+TM(s)->SIG+TM(s)+Spermine_synth->
TM+Toastrack				
AJI31452.1	BF28_3762	Bacillus cereus E33L	bacteria>firmicutes	ABC-ATPase->TM(s)->TM+Toastrack->
AAM36414.1	XAC1545	Xanthomonas citri pv citri str 306	bacteria>proteobacteria>gammaproteobacteria	GNTR-HTH->ABC-ATPase->TM(s)->TM+Toastrack->SIG+DUF2884->
AFK03672.1	Emtol_2536	Emticicia oligotrophica DSM 17448	bacteria>FCB_group>bacteroidetes	SIGMA-Factor->anti-sigma-ZF+TM+HEAT->TM+Toastrack->TM+Toastrack->
TM+Toastrack+CASPASE				
AFY83227.1	Oscil6304_3666	Oscillatoria acuminata PCC 6304	bacteria>cyanobacteria	TM+Toastrack+CASPASE->
ZnR+DUF2089-HTH+SHOCT-like				
ADE70705.1	BMQ_3692	Bacillus megaterium QM B1551	bacteria>firmicutes	ZnR+DUF2089-HTH+SHOCT-like->SHOCT-like+X->
ZnR+PspC				
ABC83427.1	Adeh_3661	Anaeromyxobacter dehalogenans 2CPC	bacteria>proteobacteria>deltaproteobacteria	ZnR+PspC->

More information available on our webapp.

Supplementary Text

1. Domain definitions

1.1. LiaI-LiaF-TM and Toastrack domains

To best characterize the LiaIGF proteins, we used PSI-BLAST searches from the three sub-sequences of the full-length proteins, N-terminal TM region of LiaF, the C-terminus globular domain (DUF2154) of LiaF, and the globular domain in LiaG (DUF4097), followed by structure-informed sequence alignment. These analyses revealed that LiaI and LiaG bear remarkable similarities to the N-terminal TM and C-terminal globular regions of the LiaF protein, respectively. We discovered that the globular domains of these LiaG–LiaF proteins are homologs of each other and that the profiles detected by Pfam in this region, DUF2154, DUF4097, and DUF2807, can be unified into a single domain called "**Toastrack**" that has a single-stranded right-handed beta-helix fold with a unique N-terminal 7-stranded region that displays a complex intertwining of strands (PDB: 4QRK; Toastrack_N, Pfam: PF17115 only has this unique N-terminal region) [**Fig. 1A**; **Table S1**]. Likewise, the homology between the 4TM (four TM) regions of LiaI and the N-terminal domain DUF2157 of LiaF led us to rename the 4TM region as '**LiaI-LiaF-TM**' [**Fig. 1B**; **Table S1**]. Thus, the results of our analyses define two new domains: LiaI-LiaF-TM and Toastrack.

1.2 PspM and PspN domains

PspM comprises two TM regions and no other distinct domains. PspN contains a short domain at the C-terminus, DUF3046, and a yet uncharacterized N-terminal domain, which we now call **PspN_N**. We found that DUF3046 is α-helical with highly conserved threonine and cysteine residues that might be required for its function.

To further characterize the DUF3046 homologs, we used nucleotide sequences rather than translated open reading frames (ORFs), followed by sequence alignment analysis [**Fig. 1B**]. We found that the DUF3046 domain, which is widespread across Actinobacteria, is more similar to the short downstream protein, Rv2738c, than to the C-terminus of the fourth member of the *M. tuberculosis* operon, PspN (encoded by *Rv2742c*).

1.3 PspAA and PspAB domains

The PspA neighborhood analysis identified a new component in the proximity of PspA, which is a protein containing a novel trihelical domain (with absolutely conserved R and D) present in Euryarchaeota, Thaumarchaeota, Actinobacteria, Chloroflexi, Firmicutes, and a few Alpha- and Gammaproteobacteria). This protein occurs in a two-gene cluster with PspA [**Fig. 3**; **Table S3**; *e.g.,* MA_1460; AAM04874.1, Methanosarcina]. This domain mostly occurs by itself but is occasionally fused to an N-terminal PspA in Actinobacteria and Chloroflexi [**Fig. 3**; **Table S3**; *e.g.,* ACU53894.1, Acidimicrobium]. We call this domain **PspAA** (for **PspA-associated domain A**; **Table S2**; web app). In a few bacterial and archaeal lineages, the PspA–PspAA dyad co-occurs with another dyad comprising a membrane-associated Metallopeptidase and a protein with a novel domain, which we termed **PspAB** (for **PspA-associated domain B**, AAZ55047.1, Tfu_1009 Thermobifida) [**Fig. 3**; **Table**

S2; web app]. This predicted operon occasionally contains a third gene coding for a SHOCT-like bihelical domain-containing protein in various bacterial and archaeal lineages [**Fig. 3**; **Table S3**; ABW11964.1, *Frankia*; AKB54760.1, *Methanosarcina*; AKX93460.1, *Moorella*].

2. Novel PSP associations

2.1 PspA/Snf7 domain architectures

A very small fraction of PspA homologs shows variation in their domain architecture (proteins that contain fusions with PspA instead of carrying PspA alone). For example, cyanobacterial PspA homologs show some interesting variations: a few have dyads or triads of PspA, either as repeated domains within a polypeptide or a predicted operon with multiple copies of PspA-containing genes [**Fig. 3**; *e.g.,* BAG06017.1], while others carry an additional hydrolase domain of NlpC/P60 superfamily at the N-terminus that is predicted to catalyze the modification of phosphatidylcholine, thus altering membrane composition [**Fig. 3**; **Table S3**; AFZ52345.1;(1)]. We also find a novel fusion of PspA with **PspAA** in Actinobacteria (ACU53894.1, *Acidimicrobium*; defined in the section on PspAA below). Similar to the PspA homologs, a search for the related superfamily, Snf7, revealed minimal variation in domain architecture, with occasional fusions (<5%) found only in eukaryotes [**Fig. 2B**]. Some Actinobacteria, such as *Mycobacteroides abscessus*, have an Snf7 homolog [CAM62382.1, **Fig. 2B**] fused to an RND-family transporter member. The latter transports lipids and fatty acid and is flanked by two genes encoding the *Mycobacterium*-specific TM protein with a C-terminal Cysteine-rich domain (2).

2.2 Vps4 and AAA$^+$-ATPases

One or more copies of an *snf7* gene [*e.g.,* OLS27540.1; **Table S3**] and a gene for the **VPS4-like AAA$^+$-ATPase** (with an N-terminal MIT domain and C-terminal oligomerization domain; **Table S2**) are known to occur together in Archaea; they define the core of an ESCRT complex (3). However, we observed some diversity between different archaeal lineages. For example, the Asgardarchaeota contain a genomic context that is most similar to eukaryotes. This archaeal context is composed of the Vps4 AAA$^+$-ATPase and Snf7-encoding genes along with an ESCRT-II gene that encodes a protein with multiple winged helix-turn-helix (wHTH) domains (4). In Crenarchaeota, Snf7 and the Vps4 AAA+-ATPase are encoded in a distinct three-gene operon, which contains a gene coding for a CdvA-like coiled-coil protein with an N-terminal PRC-barrel domain implicated in archaeal cell division (5). In this case, the Snf7 domain is fused to a C-terminal wHTH domain, which might play a role equivalent to the ESCRT-II wHTH domain. These operons may be further extended with additional copies of Snf7 genes and other genes coding for a TM protein and an ABC ATPase. We also observed that a related VPS4-like AAA$^+$-ATPase was transferred from Archaea to Bacteria and is found in Cyanobacteria, Bacteroidetes, Verrucomicrobia, Nitrospirae, and Planctomycetes (*e.g.,* ACB74714.1, Opitutus; **Table S3**). In these operons, the *snf7* gene is displaced by an unrelated gene coding for a larger protein with TPR repeats followed by a 6TM domain, again suggesting a membrane-proximal complex.

Our analysis also showed that the bacterial PspA (*e.g.,* AEY64321.1, Clostridium; **Table S3**) might occur with a distinct AAA$^+$-ATPase in various bacterial clades. The resulting protein (*e.g.,* AEY64320.1, Clostridium) has two AAA$^+$-ATPase domains (*e.g.,* CKH37208.1, *Mycolicibacterium*) in the same polypeptide, with the N-terminal version being inactive. This gene dyad also occurs with either a previously unidentified membrane-anchored protein with a divergent Snf7 domain (OGG56892.1; **Table S3**) and other coiled-coil or α-helical domain-containing proteins. Both PspA and the membrane-associated Snf7, along with the AAA$^+$-ATPase, may occur in longer operons with other genes coding for an ABC-ATPase, an ABC TM permease, and a solute-binding protein with PBPB and OmpA domains [*e.g.,* OGG56892.1; **Table S3**].

2.3 PspA with PspM or Thioredoxin

The association of ClgR-HTH with PspAM is also confined to this RsmP family, suggesting that these are also determinants of the rod-shaped morphology of the cell. The PspN presence in the immediate operon of ClgR-HTH–PspAM (containing ClgR, PspA, PspM) is limited to a few mycobacteria (CCP45543.1, *M. tuberculosis* H37Rv), which have an N-terminal PspN_N (as defined below) and C-terminal DUF3046. The remaining ClgR-HTH–PspAM operons lack the fused PspN_N–DUF3046 protein and instead contain only the ancestral DUF3046 located three genes downstream (ABK71106.1, *Mycolicibacterium smegmatis*). The duplicated DUF3046 domain forms the intact ClgR-HTH–PspAMN operon only in the *M. tuberculosis* complex (6, 7). The presence of the same family of thioredoxin with a different family of PspA (typically, two copies) in Cyanobacteria suggests that the thioredoxin homolog is involved in a similar redox activity to control PspA [AFZ14666.1, Crinalium; **Fig. 3**; **Table S3**].

2.4 Novel contexts containing Toastrack

Toastrack and TM domains

In most homologs, we find that Toastrack domains are fused to N-terminal single or multi-TM domains such as PspC, LiaI-LiaF-TM, HAAS, SHOCT, strongly suggesting that the Toastrack domains are predominantly intracellular with N-terminal membrane tethers [**Fig. 4, 5**; **Table S4**]. In Cyanobacteria, we find variable multidomain proteins with an N-terminal TM anchor followed by a region containing the Toastrack domain flanked by immunoglobulin (Ig) and one or more catalytic domains such as a fringe-like glycosyltransferase or a caspase-like thiol peptidase [**Fig. 4**; **Table S4**; AFY83227.1, *Oscillatoria*; **Table S2**]. Further, in several architectures, the N-terminal TM regions fused to the Toastrack domain are replaced by at least two variants of the bihelical SHOCT (*e.g., Bacillus subtilis yvlB*, CAB15517.1) [**Fig. 4, 5**; **Tables S1** and **S4**]. We call these variants **SHOCT-like** domains to distinguish them from the classical SHOCT domain, as these include a domain partly detected by the Pfam **DUF1707** (8) model and another that has not been detected by any published profile. The SHOCT and related domains are fused to disparate domains and are typically found at the N- or C-termini of proteins.

Toastrack and transcription factors

We also discovered several conserved genomic contexts containing Toastrack, with likely roles in membrane-linked stress response: The first of these found across diverse bacterial lineages contains a core of four genes coding for i) a sigma factor, ii) a receptor-like single TM protein with an intracellular anti-sigma-factor zinc finger (zf-HC2, PF13490 in Pfam) and extracellular HEAT repeats, iii) one or two membrane-anchored Toastrack-containing proteins (AFK03672.1 Emticicia; **Table S4**), and iv) a previously uncharacterized protein with hits to the Pfam model DUF2089. We found that this Pfam model **DUF2089** can be divided into an N-terminal ZnR, central HTH, and C-terminal SHOCT-like domains (ADE70705.1, *Bacillus*) [**Fig. 4**; **Tables S2** and **S4**]. In a few of these operons, the membrane anchor of the Toastrack domain is a LiaI-LiaF-TM domain [**Fig. 5**; **Table S4**]. Variants of this system include additional genes coding for a protein with a LiaI-LiaF-TM domain fused to an N-terminal B-box domain (*e.g.,* ACO33311.1, *Acidobacterium*) or a PspC protein (*e.g.,* OGF50123.1, *Candidatus Firestonebacteria*) [**Fig. 5**; **Table S4**]. We propose that this three-gene system functions similarly to the classical *lia* operon in transducing membrane-associated signals to a transcriptional output affecting a wide range of genes via the sigma factor.

Similarly, an operon observed predominantly in various Proteobacteria and Bacteroidetes couples a protein with a membrane-anchored Toastrack domain (typified by AAM36414.1, *Xanthomonas*) with genes coding for an ABC-ATPase, a permease subunit, and a **GNTR-HTH** transcription factor with distinct C-terminal α-helical domain and another [**Fig. 5**; **Tables S2** and **S4**]. These operons also code for a previously uncharacterized protein matching the Pfam DUF2884 model. We show that these proteins are membrane-associated lipoproteins (*e.g.,* AJI31452.1, *Bacillus*), which might function as an extracellular solute-binding partner for the ABC-ATPase and permease components. A comparable operon found in Actinobacteria replaces the **GNTR-HTH** transcription factor with a ribbon-helix-helix (**RHH**) domain protein. In some Actinobacteria, the Toastrack domain encoded by the operon is fused to a SHOCT-like domain and is encoded adjacent to genes specifying a two-component system (CCP43715.1, *Mycobacterium*) or a transport operon (CAB88834.1, *Streptomyces*) [**Fig. 5**; **Table S4**]. These operons with the Toastrack domains are likely to couple transcriptional regulation to the sensing of membrane-proximal signal and transport [**Fig. 5**; **Table S4**]. The GNTR-HTH and RHH operons in these systems are likely to function as transcriptional regulators analogous to PspF and ClgR transcription factors from classical PSP systems.

References

1. Anantharaman V, Aravind L. 2003. Evolutionary history, structural features and biochemical diversity of the NlpC/P60 superfamily of enzymes. Genome Biol 4:R11.
2. Brosch R, Gordon SV, Marmiesse M, Brodin P, Buchrieser C, Eiglmeier K, Garnier T, Gutierrez C, Hewinson G, Kremer K, Parsons LM, Pym AS, Samper S, van Soolingen D, Cole ST. 2002. A new evolutionary scenario for the Mycobacterium tuberculosis complex. Proc Natl Acad Sci U S A 99:3684–3689.
3. Wollert T, Wunder C, Lippincott-Schwartz J, Hurley JH. 2009. Membrane scission by the ESCRT-III complex. Nature 458:172–177.
4. Zaremba-Niedzwiedzka K, Caceres EF, Saw JH, Bäckström D, Juzokaite L, Vancaester E, Seitz KW, Anantharaman K, Starnawski P, Kjeldsen KU, Stott MB, Nunoura T, Banfield JF, Schramm A, Baker BJ, Spang A, Ettema TJG. 2017. Asgard archaea illuminate the origin of eukaryotic cellular complexity. Nature 541:353–358.
5. Kaur G, Iyer LM, Subramanian S, Aravind L. 2018. Evolutionary convergence and divergence in archaeal chromosomal proteins and Chromo-like domains from bacteria and eukaryotes. Sci Rep 8:6196.
6. Datta P, Ravi J, Guerrini V, Chauhan R, Neiditch MB, Shell SS, Fortune SM, Hancioglu B, Igoshin OA, Gennaro ML. 2015. The Psp system of Mycobacterium tuberculosis integrates envelope stress-sensing and envelope-preserving functions. Mol Microbiol 97:408–422.
7. Ravi J, Anantharaman V, Aravind L, Gennaro ML. 2018. Variations on a theme: evolution of the phage-shock-protein system in Actinobacteria. Antonie Van Leeuwenhoek 111:753–760.
8. Eberhardt RY, Bartholdson SJ, Punta M, Bateman A. 2013. The SHOCT domain: a widespread domain under-represented in model organisms. PLoS ONE 8:e57848.
